# Does incidental pride increase competency evaluation of others who appear careless? Discrete positive emotions and impression formation

**DOI:** 10.1371/journal.pone.0220883

**Published:** 2019-08-08

**Authors:** Toshiki Saito, Kosuke Motoki, Rui Nouchi, Ryuta Kawashima, Motoaki Sugiura

**Affiliations:** 1 Institute of Development, Aging and Cancer, Tohoku University, Sendai, Japan; 2 Japan Society for the Promotion of Science, Tokyo, Japan; 3 Department of Food Management, Miyagi University, Sendai, Japan; 4 Smart-Aging Research Center, Tohoku University, Sendai, Japan; 5 International Research Institute of Disaster Science, Tohoku University, Sendai, Japan; Ghent University, BELGIUM

## Abstract

Emotion plays important and diverse roles across various social relations. Although the social functions of emotion have attracted increased attention, the effects of positive emotions such as pride on impression formation remain poorly understood. Drawing on social projection theory, this study examined how incidental experiences of pride influenced the impressions of those who made a blunder, along with two other characteristics: the person’s warmth and competence. Participants were designated randomly to receive inductions of pride, awe, or a neutral emotion. Subsequently, they were asked to indicate their own impression of a person who had made a blunder and to rate their overall sense of that individual’s warmth and competence. A laboratory experiment recruiting university students (Study 1, N = 79) demonstrated that pride, a positive emotion elicited by a self-relevant achievement, led to higher competency evaluations of others. However, a pre-registered online experiment in middle-aged adults (Study 2, N = 108) failed to replicate the effects of pride on competency evaluations of others. Furthermore, another pre-registered online experiment in younger adults (Study 3, N = 290) did not show successful manipulation of incidental emotions. These results suggest that strictly controlled experimental settings that induce robust incidental emotions might be better for demonstrating a strong pride effect on the evaluation of others.

## Introduction

Our thoughts about others and determination of their traits are fundamental behaviors occurring on a daily basis [1]. To infer another’s character is an important skill that one must have to develop relations with others and to avoid conflict [2]. Therefore, by exploring both appearances and internal states, many studies have been undertaken to investigate factors that affect impression formation (e.g. [3]).

In past decades, research has established that humans perceive traits along two dimensions: warmth and competence [4, 5]. The warmth dimension relates to traits such as friendliness and morality, whereas the competence dimension relates to one’s ability and intelligence (e.g., status and self-esteem). Understanding these traits correctly provides important benefits. For instance, our judgment of warmth enables us to avoid potential conflict, whereas accurate judgment of competence is necessary for efficient cooperation with others.

Although earlier studies showed that same-valence emotions such as anger and fear differently influence judgment and decision-making [6], whether such same-valence emotions also influence impression formation remains unknown. Especially, the functions of positive discrete emotions such as pride remain unknown. The present research specifically examined whether incidental emotion which is a situational change of emotional states produced by one setting, affects impression formation. Particularly, we examined how incidental pride and a blunder influence two other dimensions: warmth and competence. Many researchers have demonstrated that the valence of affective states can influence how people identify their own perceptions or decisions. Schwarz and Clore [7] reported that people misattribute their emotional state (e.g., caused by a sunny or rainy day) to ratings of life satisfaction when they are unaware of the reasons for their current mood. Based on this finding, they developed a model known as “feeling-as-information,” which states that people attend to their current feelings as a source of information in forming judgments [8]. Forgas and Bower [9] also studied the effects of emotion on impression formation. In their study, participants experiencing a positive emotional state formed more favorable impressions than participants experiencing a negative mood. However, no report describes a study investigating the manner in which discrete positive emotions influence impression formation.

Emotional states profoundly influence the manner by which information is processed. The valence of any given emotion directs processing to assimilative and top-down, or to accommodative and bottom-up. Assimilation implies that new information is understood by referring to existing cognitive schemas, whereas accommodation suggests that existing cognitive schemas and information are modified in accordance with new information. Earlier studies have revealed that positive emotional states promote assimilative processing; conversely, negative emotional states tend to facilitate accommodative processing [10]. With respect to the affective influence on social cognition, people in positive emotional states tend to judge others based on their internal states more so than people in negative emotional states.

Although most early studies investigating the effects of emotional states on cognition have specifically examined mood (positive or negative feelings), recent knowledge suggests that even if the emotional valence remains unchanged, each emotion has different effects on subsequent judgment (for review, [11]). For instance, although both pride and happiness have a positive valence, only happiness decreases self-control [12].

Pride is a positive, self-conscious emotion arising from achievements gained through one’s own ability or effort [13, 14]. For example, if one achieves success for a long-term goal, then one would feel pride. Pride has a crucially important role in many domains of psychological functioning. Indeed, pride facilitates one’s motivation for attaining status and self-acceptance [15, 16]. Pride is also well known to be able to create feelings of “an emerged self” or high self-esteem [17, 18]. For instance, participants who experienced pride were more likely to take on a dominant role in a group problem-solving task, but they incur the cost of being perceived as less likable by others in the group [19].

Awe is another positive emotion. It arises from the perception of something much larger than oneself [20]. Although awe is a positive emotion, the experience results in a diminished sense of the individual self [21]. For instance, Piff et al. [22] demonstrated that inducing awe in study participants caused them to feel small and insignificant.

Given that positive emotional states promote assimilation, both pride and awe are expected to facilitate a judgmental heuristic such as social projection for impression formation. Social projection is a judgmental heuristic by which people project their own internal state on their judgment of others [23]. Therefore, pride is associated with both greater self-esteem and enhanced evaluation of others’ competence, although awe elicits a diminished sense of self and decreased competency evaluation of others. We hypothesized that incidental pride enhances the competency evaluation of others, but that awe diminishes it.

Additionally, we examine how the occurrence of a blunder influences incidental emotion effects on impression formation. Such displays of human frailty might increase social projection by providing a sense of similarity to oneself. For example, a person who makes a blunder (e.g. spilling a cup of coffee over oneself) might be regarded as more human and approachable: the phenomenon is known as the pratfall effect [24, 25]. This is a key indicator of social projection (similarity between oneself and others) [26]. Indeed, social projection is stronger when people judge ingroups than when they judge outgroups [27]. Therefore, the possibility exists that the incidental emotion effects on competency evaluation are amplified by a blunder because of social projection. We predicted that a blunder would increase the competency evaluation of others in a setting of pride.

## Study 1

This study explored how the incidental emotions of pride and awe, as well as the occurrence of a blunder, influence impression formation. Incidental pride is expected to engender a higher competency evaluation of others, although the opposite is expected to occur with awe. Internal competence influences the degree of positive emotion directed to others during impression formation (H1). The incidental emotion affecting impression formation is amplified by a blunder, as facilitated by social projection (H2).

## Methods

### Participants

In this study, 79 Tohoku University undergraduates and graduates (44 men, 35 women) participated. The mean age of the participants was 21.22 (SD = 1.95). Each received $10 for their participation. We decided on the sample size based on an earlier study that examined the emotional state effect on person perception [9]. That study recruited 26 participants per condition and found a large effect size. Therefore, we gathered data from 25–27 participants for each condition. This sample size met a critically minimum requirement that there be at least 20 observations per conditions to avoid failure at detecting most effects [28]. After this experiment, however, we conducted a replication study with a larger sample, following Simmons and his colleagues’ opinion that a study with fewer than 50 per cell has insufficient power unless some evidence exists to the contrary [29]. We obtained written informed consent from each participant.

### Design

This study took the form of a 3 (Manipulation of emotion: Pride vs. Awe vs. Neutral) × 3 (a degree of blunder: big vs. small vs. none) × 3 (Impression items: warmth vs. competence vs. attractiveness) mixed-model design, with manipulation of emotion as a between-subjects factor. Both the degree of blunder and impression items served as a within-subjects factor. This study was approved by the ethics committee of the School of Medicine at Tohoku University (approval number: UMIN000025712) and was conducted in accordance with the Declaration of Helsinki.

### Emotion manipulation

To manipulate the participants’ emotional states, we used an emotion recall task [22]. The task had two parts: first, participants were asked to list events associated with pride or awe, or daily events associated with no emotion. Pride was defined as “Satisfaction derived from one’s own achievement. For instance, being accepted to a university, being awarded a prize, winning a race or accomplishing an important task might cause you to feel pride.” Awe was defined as “A feeling of respect mixed with fear of the sublime. For instance, viewing a beautiful natural scene such a starry sky or vast ocean, or a view from high place might cause you to feel awe.” Subsequently, participants were asked to choose an event that elicited their greatest emotion. They were then instructed to write down the situation (when, where and with whom) and their feelings in full detail. Each part took about 3 min. Before and after the manipulation, they were asked to rate their current emotional state (pride, awe, valence and arousal) on a seven-point Likert-type scale (pre-rating), where 1 denoted “not at all” and 7 denoted “very much”.

### Blunder description

Participants read fictitious descriptions similar to those used in an earlier study [30]. Along with the degree of blunder, there were three descriptions of people named Mr./Mrs. A, B, and C. Each description was as follows: (1) “Mr./Mrs. A is such a smart person. One day, he carelessly tipped over a cup and spilled coffee all over himself.” (big blunder); (2) “Mr./Mrs. B is such a smart person. One day, he carelessly tipped over a cup and spilled coffee all over the floor.” (small blunder); and (3) “Mr./Mrs. C is such a smart person. One day, he was accidentally bumped by someone and spilled coffee all over himself.” (non-blunder). We used two intensities of blunder to rule out the possibility that a consequence of an event, but not the blunder, influenced the effect of emotions. Strong effects of emotion would be seen in targets with either big or small blunder if a mistake itself caused social projection. However, if a consequence of the events, such as spilling coffee all over oneself in this case, caused social projection, effects would be observed in targets with either a big blunder or non-blunder.

### Procedure

We pseudo-randomly allocated participants into three groups (pride, awe, and neutral). The pride and awe groups included 27 participants (15 women in pride, 11 women in awe). The neutral group included 25 participants (9 women). Before obtaining informed consent from each participant, each was told that they would perform two independent tasks.

In the first phase, they performed the emotion recall task. In the next phase, participants read the three pratfall descriptions and rated their impressions using three seven-point semantic differential scales (incompetent—competent, cold—warm, unattractive—attractive). Afterwards, participants were debriefed and thanked for their participation. The order of descriptions and scales was not counterbalanced because there was no order effect in earlier studies [31]. Participants rated targets in the following order: targets with a small blunder, big blunder, and non-blunder. They rated items in the order of competence, attractiveness, and warmth.

### Statistical analysis

We performed a Hyunh–Feldt corrected mixed-measures analyses of variance (ANOVA) to assess the validity of emotion manipulation. The design was 3 (Emotion Manipulation: pride, awe, neutral) × 4 (Emotional state: pride, awe, valence, arousal). The dependent value was the change in score of emotional rating: the post-rating was subtracted from the pre-rating.

To assess the emotion’s effect on impression formation, we applied a mixed-measure ANOVA. The design was 3 (emotion manipulation) × 3 (degree of blunder: big blunder, small blunder, non-blunder) × 3 (impression judgment: warmth, competence, attractiveness). The dependent value was the impression rating.

After we found a significant main effect, we conducted multiple testing for interpretation. In doing so, we applied the modified sequentially rejective Bonferroni method. We conducted multiple testing six times. Confidence intervals for both Cohen’s d and partial eta squared were calculated to assess the accuracy of the effect size using a website [32]. All statistical analyses were conducted using R software [33]. All ANOVA and subsequent multiple tests were done using anovakun, a function of R software.

## Results

### Emotion manipulation check

We first conducted an analysis of the emotion manipulation check. We found a significant interaction effect between emotion manipulation and the emotion state (*F* (5.96, 220.46) = 14.09, *p* < .0001, *η*_*p*_^*2*^ = 0.276, 90% confidence interval or CI = [0.18, 0.33]). From post-hoc analyses, we found significant simple main effects of emotion manipulation in both ratings of pride and awe (*Fs* (2, 74) = 9.32, 39.52, *ps* = .0002, .0001, *η*_*p*_^*2*^*s* = 0.201, 0.517, 90% CIs = [0.07, 0.31], [0.37, 0.60]). However, we did not find a simple main effect in valence or arousal ratings (*Fs* (2, 74) = 2.46, 3.07, *ps* = .092, .053, *η*_*p*_^*2*^*s* = 0.006, 0.077, 90% CIs = [0.00, 0.15], [0.00, 0.17]). Multiple comparisons demonstrated that the participants in the pride condition reported significantly higher pride ratings (M = 0.963, SD = 1.22) than participants in either the awe or neutral condition (M = 0.00, -0.39, SD = 1.30, 0.84; *ts*(74) = 3.07, 4.14, *ps* = .003, .0001, *ds* = 0.76, 1.26, 95% CIs = [0.21, 1.31], [0.68, 1.88]). Participants in the awe condition also reported significantly higher awe ratings (M = 2.11, SD = 1.40) than participants in the pride or neutral condition (M = -0.07, -0.17, SD = 0.83, 0.78; *ts*(74) = 7.64, 7.66, *ps* < .000, .000, *ds* = 1.88, 2.00, 95% CIs = [1.24, 2.53], [1.31, 2.65]). Nevertheless, ratings of valence and arousal were not significantly different among the three emotion manipulation conditions (see Table 1). From these results, we confirmed that our emotion manipulation was successful.

**Table 1:** Average changes of emotional rating scores (Study 1).

	Emotional states							
Group	Pride		Awe		Valence		Arousal	
Pride	0.96	(1.22)	-0.07	(0.83)	0.41	(1.42)	1.22	(1.76)
Awe	0.00	(1.30)	2.11	(1.40)	-0.15	(0.86)	0.63	(1.33)
Neutral	-0.39	(0.84)	-0.17	(0.78)	-0.22	(0.95)	0.22	(1.13)

Standard deviations are shown in parentheses

### Effects of incidental emotion

To assess the effect of emotional states on the impression ratings, we conducted a three-way ANOVA analysis. It is noteworthy that we found a significant interaction between the manipulation of emotion and impression item (*F* (3.34, 126.77) = 3.14, *p* = .024, *η*_*p*_^*2*^ = 0.08, 90% CI = [0.01, 0.13]). Post hoc-analyses, revealed significant simple main effects of emotion manipulation for both competency and attractiveness judgments (*Fs* (2, 76) = 4.50, 3.16, *ps* = .014, .048, *η*_*p*_^*2*^*s* = 0.106, 0.077, 90% CIs = [0.01, 0.20], [0.00, 0.17]). This result suggests that discrete positive emotions specifically influenced impression formation.

**Fig 1:**
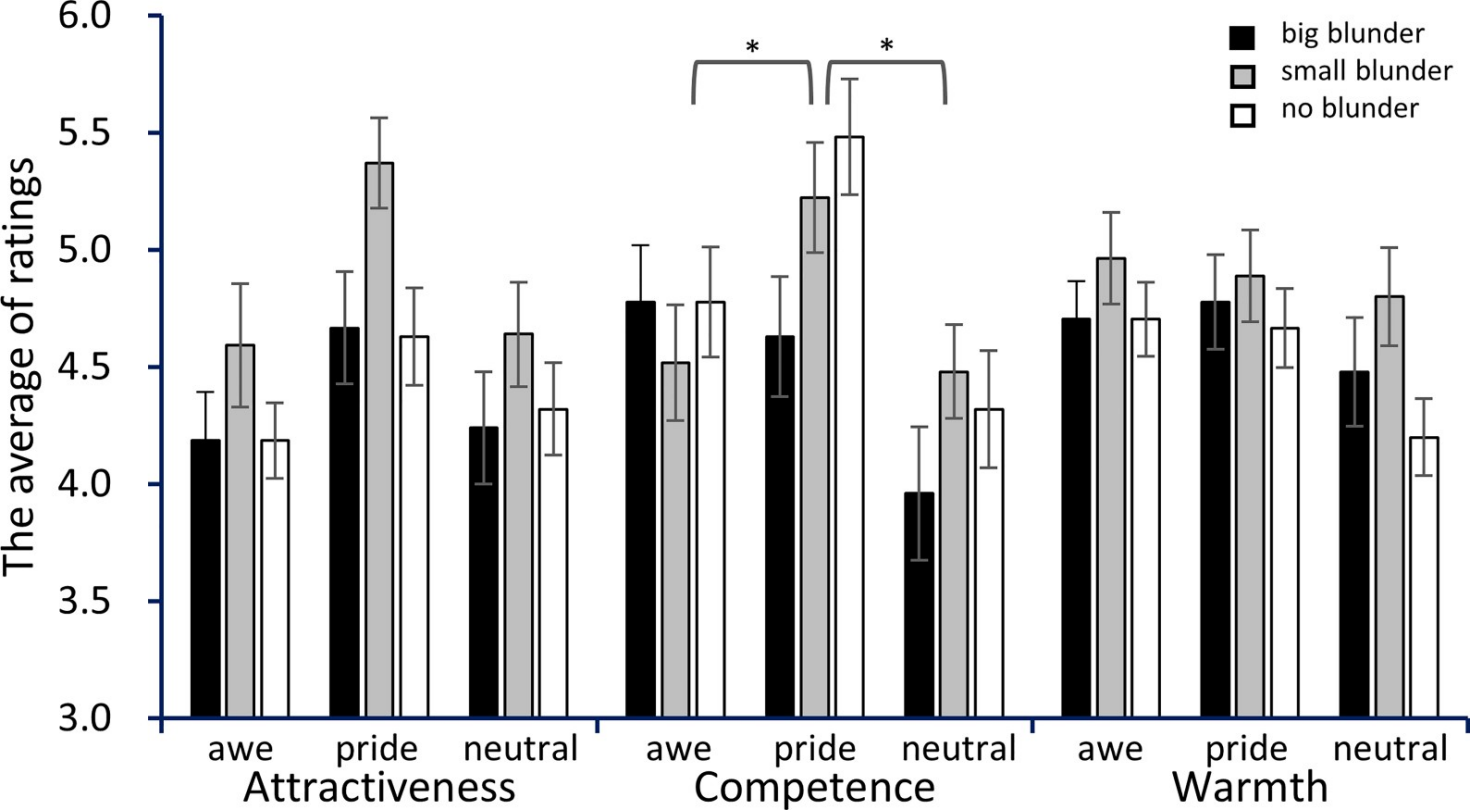
Average ratings for blunders of three types. Asterisks denote significant simple main effects of incidental emotion at time of competency evaluation (*p* < .05). Error bars represent standard errors.

To test how pride and awe influenced competency evaluation of others, we conducted multiple comparisons for our hypotheses. Results show that participants in the pride condition assigned significantly higher ratings of competency (M = 5.11, SD = 1.31) than those in the awe (M = 4.36, SD = 1.31; *t* (76) = 2.44, *p* = .024, *d* = 0.57, 95% CI = [0.03, 1.11]) or neutral conditions (M = 4.25, SD = 1.24; *t* (76) = 2.72, *p* = .024, *d* = 0.67, 95% CI = [0.11, 1.23]). By contrast, those in the awe condition showed neither a promoted nor reversed effect on perceived competence compared with neutral condition (*t* (76) = 0.33, *p* = .741, *n*.*s*., *d* = 0.08, 95% CI = [-0.46, 0.63]). Given the theoretical background, the results of pride are consistent with our expectation. However, the result of awe is inconsistent with our expectations (Fig 1).

To test how pride and awe also influenced attractiveness evaluation of others, we conducted multiple comparisons. Results show that participants in pride also gave higher ratings of attractiveness (M = 4.89, SD = 1.15) than those in the awe (M = 4.33, SD = 1.12; *t* (76) = 2.31, *p* = .071, *d* = 0.43, 95% CI = [-0.05, 1.03]) or neutral conditions (M = 4.40, SD = 1.10; *t* (76) = 1.99, *p* = .071, *d* = 0.44, 95% CI = [-0.11, 0.98]). However, those in the awe condition showed neither promoted nor reversed effects on perceived competence compared with the neutral condition (*t* (76) = 0.27, *p* = .787, *n*.*s*., *d* = 0.06, 95% CI = [-0.61, 0.48]). Although these differences were marginally significant but not significant, the patterns of emotional effects on impression are similar to results of competency evaluation.

No interaction was apparent between the manipulation of emotion and the degree of blunder (F (3.82, 145.18) = 0.48, *p* = .745, *η*_*p*_^*2*^ = 0.01, 90% CI = [0.00, 0.29]), nor did we find any three-way interaction (F (4.49, 117.14) = 1.46, *p* = .184, *η*_*p*_^*2*^ = 0.04, 90% CI = [0.00, 0.10]). These results suggest that the emotion effect was unmediated by the degree of blunder. A full report of these statistical analyses is presented in *Supplementary Materials*, S1 File.

### Blunder effect

A significant effect was found for the degree of blunder (*F* (1.91, 156.29) = 12.13, *p* < .001, *η*_*p*_^*2*^ = 0.14, 90% CI = [0.05, 0.21]), indicating that participants rated a target with a small blunder favorably (M = 4.83, SD = 1.15), and significantly higher than either a target with no blunder (M = 4.59, SD = 1.09; *t* (76) = 2.34, *p* = .022, *d* = 0.21, 95% CI = [-0.10, 0.53]) or a target with a big blunder (M = 4.37, SD = 1.21; *t* (76) = 5.54, *p* < .0001, *d* = 0.39, 95% CI = [0.07, 0.70]). Results also show significant interaction between the degree of blunder and impression items (*F* (3.47, 263.83) = 11.64, *p* < .001, *η*_*p*_^*2*^ = 0.13, 90% CI = [0.07, 0.19]). Post-hoc analyses indicated that the target with small blunder was perceived favorably in almost all items. A full report of these statistical analyses is described in *Supplementary Materials*, S1 File.

## Discussion

After experiencing incidental pride, awe, or neither, participants rated their impressions about someone who had just made a blunder. Results show that incidental pride biases the competency evaluation of others. Participants who experienced pride provided higher ratings of competence than in other conditions. Although it is marginally significant, they also provided higher ratings of attractiveness than in other conditions. The degree of blunder did not amplify the pride effect on impression formation. Warmth ratings showed no difference among conditions.

## Study 2

In the first study, we observed that incidental pride biased competency evaluation of others. However, two important limitations were the small sample size and the fixed order of descriptions and scales. According to Simmons and his colleagues, 20 participants-per-cell is considered a critical minimum, not a sufficient number for acquiring sufficient power [28, 29]. The fixed order might also have affected the results. For example, the effects of emotion might be diminished in a later target compared to an earlier target by the passage of time. To overcome these issues, we conducted a replication online study. This study was pre-registered with Open Science Framework (https://osf.io/57utk).

## Methods

### Participants

The second study examined 108 online participants (57 men, 51 women; mean age 40.48, SD = 9.81). Participants received $2 for their participation. We calculated the sample size with effect size of *f* = 0.31145 derived from the first study. Results of power analyses conducted with software (GPower 3.1) suggest that a sample size of 103 participants is sufficient to detect main effects of incidental emotion. For this analysis, we set the power at 80%. Results show that the pride group included 33 participants (15 women). The awe group included 37 participants (17 women). The neutral group included 38 participants (19 women).

### Design

The materials and procedure for the second study were identical to those of the first study, except as noted. We collected data through Qualtrics (https://www.qualtrics.com/jp/) from Japanese online participants of Lancers (https://www.lancers.jp/), a cloud sourcing website in Japan. For the current study, we randomized the order of descriptions and scales and added the following ratings to evaluate mediational factors, competency evaluation related to self (1, incompetent– 7, competent), and perceived similarity of targets (1, dissimilar– 7, similar). Participants answered a self-esteem questionnaire [34].

### Statistical analysis

We applied Hyunh–Feldt corrected mixed-measures analyses of variance (ANOVA) to assess the validity of emotion manipulation. This analysis was identical to that conducted for the first study.

We performed analyses of covariance (ANCOVA) to assess the validity of emotion manipulation. An independent variable was emotion manipulation (coded as pride, 0; awe, 1; neutral, 2). The dependent value was competency evaluation. We included a self-esteem score as a covariate because self-esteem was apparently related strongly to self-competency evaluation.

## Results

### Emotion manipulation check

We first conducted an analysis of the emotion manipulation check, which revealed a significant interaction effect between emotion manipulation and emotion states (*F* (5.60, 293.93) = 2.36, *p* < .033, *η*_*p*_^*2*^ = 0.043, 90% CI = [0.00, 0.07]). Post-hoc analyses revealed significant simple main effects of emotion manipulation in both ratings of pride and awe (*Fs* (2, 105) = 4.01, 9.39, *ps* = .021, .0002, *η*_*p*_^*2*^*s* = 0.07, 0.15, 90% CIs = [0.01, 0.15], [0.05, 0.25]). However, no simple main effect was found in valence and arousal ratings (*Fs* (2, 105) = 0.48, 1.97, *ps* = .620, .144, *η*_*p*_^*2*^*s* = 0.01, 0.04, 90% CIs = [0.00, 0.05], [0.00, 0.10]). Multiple comparisons revealed that participants in the pride condition reported significantly higher pride ratings (M = 0.52, SD = 1.00) than participants in either the awe or neutral condition (M*s* = 0.05, -0.03, SD*s* = 0.88, 0.68; *ts*(105) = 2.25, 2.66, *ps* = .028, .028, *ds* = 0.76, 1.26, 95% CIs = [-0.02, 0.93], [0.12, 1.07]). Participants in the awe condition also reported significantly higher awe ratings (M = 0.81, SD = 1.35) than participants in the pride or neutral condition (M*s* = -0.03, -0.18, SD*s* = 0.68, 1.01; *ts*(105) = 3.31, 4.06, *ps* = .001, .0003, *ds* = 0.76, 1.26, 95% CIs = [0.28, 1.26], [0.36, 1.30]). However, ratings of valence and arousal were not significantly different across the three emotion manipulation conditions (see Table 2). These results confirmed that our emotion manipulation was successful.

**Table 2:** Average changes in scores of emotional ratings (Study 2).

	Emotional states							
Group	Pride		Awe		Valence		Arousal	
Pride	0.51	(1.00)	-0.03	(0.68)	-0.52	(1.12)	-0.18	(1.49)
Awe	-0.03	(0.68)	0.81	(1.35)	0.59	(0.86)	0.57	(1.30)
Neutral	0.05	(0.88)	-0.18	(1.00)	0.37	(1.05)	-0.13	(1.74)

Standard deviations are shown in parentheses.

### Effects of incidental emotion

To assess emotional state effects on the impression ratings, we applied ANCOVA. Unlike the first study, no significant effect of incidental emotion was found (*F* (2, 104) = 0.86, *p* = .425, *n*.*s*., *η*_*p*_^*2*^ = 0.016, 90% CI = [0.00, 0.06]).

## Discussion

Unlike the first study, the effect of incidental pride was not replicated in the second study. We assumed that the following three differences of the replication study from the first study might underlie this failure of replication: the sample size, participant characteristics, and emotion manipulation.

First, it is unclear whether the sample size was adequate to detect the effect of pride. Although we estimated the sample size based on the results of the first study, it might be true that the estimation was inaccurate because of the small sample size of the first study. Thus, there was a risk to overlook the actual effect due to the inadequate sample size. Secondly, participant characteristics were quite varied in the second study. Although participants in the first study were undergraduate and graduate students (Mean age was 21.22, SD = 1.95), participants in the second study were much older (Mean age was 40.48 SD = 9.81). People are known to become gradually more skilled at handling their emotions [35]. Therefore, the possibility exists that incidental emotion caused by our manipulation did not persist in the evaluation task in the second study. Thirdly, the emotion manipulation situation differed. For the first study, 4–6 participants performed the emotion manipulation task simultaneously. By contrast, participants in the second study performed the task individually. Considering that the experience of emotion depends on social context [36, 37], participants in the second study might not experience sufficient intensity of emotion. Indeed, second study’s changes in score of emotional ratings (pride = 0.51, awe = 0.81) were smaller than those in the first study (pride = 0.96, awe = 2.11).

## Study 3

Study 2 did not replicate the effect of pride on competency evaluation. In study 3, we sought to replicate the effect of pride with a younger cohort, similar to the cohort in study 1. We also increased the sample size to provide sufficient power. This study was also pre-registered with Open Science Framework (https://osf.io/bq5uj).

## Method

### Participants

We gathered larger sample sizes than for the previous two studies. According to Simmons and colleagues, between-subjects studies of attenuated interactions should have at least 100 participants per cell [29]. Thus, we gathered data from 94–99 participants for each condition. In total, 290 online participants participated in this study (99 men, 191 women; mean age 27.33, SD = 2.71). Participants received $2 for their participation. The pride group included 97 participants (64 women), the awe group included 99 participants (63 women), and the neutral group included 94 participants (64 women).

### Design & statistical analysis

The materials, procedure, and statistical analysis for study 3 were identical to those for study 2.

## Result

### Emotion manipulation check

We first conducted an analysis of the emotion manipulation check. Unlike the previous two studies, study three showed no significant interaction effect between emotion manipulation and emotion states (*F* (10.66, 861.38) = 1.78, *p* = .105, *η*_*p*_^*2*^ = 0.012, 90% CI = [0.00, 0.01]). We also found no trend indicating which group felt greater pride after the manipulation task compared with the other groups (see Table 3). These results confirmed that the emotion manipulation had failed. Therefore, we terminated the statistical analysis.

**Table 3:** Average changes in emotional rating scores (Study 3).

	Emotional states							
Group	Pride		Awe		Valence		Arousal	
Pride	0.03	(1.03)	0.03	(1.08)	0.16	(1.03)	0.07	(1.17)
Awe	0.07	(0.70)	0.62	(1.29)	0.28	(0.88)	0.16	(1.26)
Neutral	-0.10	(0.98)	0.04	(0.84)	0.11	(0.94)	-0.14	(1.25)

Standard deviations are shown in parentheses.

## Discussion

Although we tried to replicate the effect of pride found in the earlier research, we failed to manipulate emotional states successfully. Therefore, we cannot reach any conclusion regarding the effect of pride based on this study. However, regarding emotion manipulation in an online situation, we speculate that our procedure for manipulating emotional states was not effective for online participants. Indeed, we also found low emotional intensity in study 2.

## General discussion

We examined how the experience of incidental pride influenced impressions of others who had made a blunder, as well as perceptions of that person’s warmth and competence. After experiencing incidental pride, awe, or neither, participants rated their impressions about someone who had just made a blunder. Results of a laboratory experiment (study 1) illustrated that incidental pride can bias the competency evaluation of others. The participants who experienced pride gave higher competence ratings, as well as slightly increased attractiveness ratings, as compared to the other conditions. However, a pre-registered online experiment with a larger sample size (study 2) failed to replicate the main findings. Participants who experienced pride (vs. awe or neutral) did not show higher ratings of others’ competence. Furthermore, an additional pre-registered online experiment in younger adults failed to show successful manipulation of incidental emotions. These findings suggest that strictly controlled experimental settings that induce robust incidental emotions might be better for observing a strong pride effect on the evaluation of others.

The results of the first study partially supported our hypothesis. Earlier reports have indicated that positive mood facilitates the evaluation of others in a more favorable light [9]. Our results demonstrated that pride, which is a positive emotion elicited by a self-relevant achievement, led to higher competency evaluations of others. Awe, a positive emotion elicited by a perception of something vast or inspirational, did not have the same effect. Although the effects of emotions on decision-making and judgment have been well studied [9, 38–41], the effects of an observer’s incidental emotions on outward perception have not been previously explored. No report of the relevant literature describes a study demonstrating that certain positive emotions facilitate specific dimensions of impression, while other emotions do not.

Although we observed the effect of pride in study 1, study 2 failed to replicate the effect. Therefore, it is important to note that the effect of pride on competency evaluations of others might be very limited. From the results of the current studies, we speculate that this effect of pride occurs under the following conditions. First, according to Ames [42], perceived similarity with a target increases the use of social projection (i.e., assumed similarity) to infer the target’s characteristics. Participants in the first study were students at Tohoku University, considered one of the most prestigious in the country. Thus, they might have felt more similarity to the targets, who were explicitly described as intelligent, than did those in the study 2. If the effect of pride is genuine, the perceived similarity between targets and perceivers might have triggered the effect. Second, the intensity of pride in study 1 was likely to have been greater than that in study 2, as the latter was conducted online. Moreover, study 3, which was also an online experiment, did not show successful emotion manipulation. From these results, it appears that the induction of emotion in an online setting was more difficult than in the strictly controlled experimental setting. Therefore, pride might be effective only under conditions that elicit appropriately intense emotion. According to Bavel and colleagues, contextual differences between original research and replication attempts, such as a different time or a different sample, are among the key factors predicting failures of reproductivity [43]. As noted above, there are contextual differences between study 1 and studies 2 and 3, especially those with different settings (laboratory vs. online). Thus, the effect of pride on competency evaluation might require a context similar to that of study 1, such as a university student sample and a controlled experimental setting.

The effect of pride that we observed in a strictly controlled experimental setting (study 1) is largely consistent with the assumed similarity bias. Assumed similarity refers to the belief that other people are similar to oneself [44]. Therefore, people tend to see others’ traits as being similar to their own. In a study by Srivastava, Guglielmo, and Beer [44], participants played a brief group icebreaker game. Next, they rated the traits of all other group members and then rated themselves. The results demonstrated a positive correlation between ratings of oneself and those of others. Considering the assumed similarity bias, participants in the pride condition might have evaluated the competence of others based on their own self-evaluation, as amplified by pride. The authors wonder why awe did not exert a similar influence if this assumption is true. One explanation might involve a sense of similarity to others. Pride is often associated with an enhanced sense of similarity to others [45]. The current study showed that all targets rated by participants were competent (i.e., strong). Therefore, the pride group might have had an enhanced sense of similarity to the targets, more so than with the awe group, which might explain why the similarity bias was apparent for one group and not the other. They might have shown a greater assumed similarity bias if the awe group had rated an incompetent target. Additional studies must be conducted to confirm this supposition. Among reports of studies examining assumed similarity, which has been treated as a stable disposition of individuals [44, 46], our study is the first specifically emphasizing the effects of variable current states.

Our results did not completely confirm the effect of blunder on impression formation because the target with a small, but not a big blunder only seemed favorable. Absence of the effects of blunder might be caused by a difference of stimuli characteristics from those of earlier studies. Actually, earlier studies used audio stimuli to which the target reacted with an anguished emotion to a blunder [24, 30]. However, our experiment only presented the blunder behavior, with no emotional reaction to the incident. The emotional reaction to the incident is expected to be important for overall impression formation.

### Study limitations and future research

This study has some important limitations. First, the result of our laboratory experiment (study 1) provided partial evidence supporting the specificity of the pride effect. A similar pattern is apparent for both competence and attractiveness ratings under the small blunder condition, probably reflecting similar effects of pride on both ratings. Indeed, a simple main effect of emotion manipulation is apparent on the attractiveness judgment. No significant effects of pride on the attractiveness judgment were found in detailed multiple comparison analysis. We inferred that pride specifically influenced the competence evaluation, but further studies must be conducted to confirm our inference.

Second, we could not replicate the effect of pride in studies 2 and 3. In study 1, the effect size of pride was interpreted as moderate. Additionally, the 95% confidence intervals for the effect sizes did not contain zero. Thus, the effect of pride that we observed in a strictly controlled experimental setting (study 1) was interpreted as significant. However, as we noted in the previous section, online emotion manipulation was not adequate to induce emotion of sufficient intensity to cause the pride effect because it was difficult to control for contextual differences (e.g., characteristics of participants and time of participation). Thus, a further study that successfully manipulates emotional states, as study 1 did, is needed to confirm the reliability of the effect of pride on competency evaluation.

Finally, we did not check directly for potential mediators such as the degree of self-esteem (e.g., self-competency evaluation) and motivations for prosocial behavior in study 1, where we observed the effect of pride. Both of these serve to mediate the effects of incidental pride on impression formation. Although we speculated that heightened self-esteem increased the competency evaluation of others, we did not test whether an individual’s self-esteem varied with emotion manipulation. Therefore, further studies must be conducted to confirm, in greater detail, whether and why pride increased the competency evaluation of others.

## Summary

The current study examined how positive emotions such as pride and awe influence impression formation. It is particularly interesting that pride, but not awe, exerted an influence. Moreover, the pride effect was specific to competency evaluation. Although it is important to be cautious about the effect of pride, given the failure to replicate in studies 2 and 3, the current research contributes to a greater understanding of the effects of incidental emotion on impression formation. This contribution differs from those obtained through past approaches, which have specifically examined the valence of emotion. The results of the current study provide the first evidence that, even if the valence remains unchanged, discrete emotions have a different effect on impression formation.

## Supporting information

S1 File A full report of the statistical analyses in Study 1. (XLSX)

S2 File Excel data file for manipulation check in Study 1. (XLSX)

S3 File Excel data file for the primary analysis in Study 1. (XLSX)

S4 File Excel data file for manipulation check in Study 2. (XLSX)

S5 File Excel data file for the primary analysis in Study 2. (XLSX)

S6 File Excel data file for manipulation check in Study 3. (XLSX)

S7 File Excel data file which participants answered in Study 3. (XLSX)
